# Methods used in adaptation of health–related guidelines: A systematic survey

**DOI:** 10.7189/jogh.07.020412

**Published:** 2017-12

**Authors:** Rima A Abdul–Khalek, Andrea J Darzi, Mohammad W Godah, Lama Kilzar, Chantal Lakis, Arnav Agarwal, Elias Abou–Jaoude, Joerg J Meerpohl, Wojtek Wiercioch, Nancy Santesso, Hneine Brax, Holger Schünemann, Elie A Akl

**Affiliations:** 1 AUB GRADE Center, Clinical Research Institute, American University of Beirut, Beirut, Lebanon; 2 Faculty of Health Sciences, American University of Beirut, Beirut, Lebanon; 3 Faculty of Medicine and Medical Sciences – University of Balamand, Balamand Al Kurah, Lebanon; 4 Department of Medicine, McMaster University, Canada; 5 Faculty of Medicine, University of Toronto, Toronto, Canada; 6 State University of New York at Buffalo (SUNY University at Buffalo), Buffalo, USA; 7 Inserm/Université Paris Descartes, Cochrane France, Hôpital Hôtel–Dieu, Paris, France; 8 Department of Health Research Methods, Evidence, and Impact, McMaster University, Hamilton, Canada; 9 Faculty of Medicine, Univeristé Saint Joseph, Beirut, Lebanon; 10 Department of Medicine, American University of Beirut, Beirut, Lebanon

## Abstract

**Background:**

Adaptation refers to the systematic approach for considering the endorsement or modification of recommendations produced in one setting for application in another as an alternative to de novo development.

**Objective:**

To describe and assess the methods used for adapting health–related guidelines published in peer–reviewed journals, and to assess the quality of the resulting adapted guidelines.

**Methods:**

We searched Medline and Embase up to June 2015. We assessed the method of adaptation, and the quality of included guidelines.

**Results:**

Seventy–two papers were eligible. Most adapted guidelines and their source guidelines were published by professional societies (71% and 68% respectively), and in high–income countries (83% and 85% respectively). Of the 57 adapted guidelines that reported any detail about adaptation method, 34 (60%) did not use a published adaptation method. The number (and percentage) of adapted guidelines fulfilling each of the ADAPTE steps ranged between 2 (4%) and 57 (100%). The quality of adapted guidelines was highest for the “scope and purpose” domain and lowest for the “editorial independence” domain (respective mean percentages of the maximum possible scores were 93% and 43%). The mean score for “rigor of development” was 57%.

**Conclusion:**

Most adapted guidelines published in peer–reviewed journals do not report using a published adaptation method, and their adaptation quality was variable.

Guideline adaptation provides an alternative to de novo guideline development bz making the process more efficient and avoiding duplication of efforts. Given that guidelines produced in one setting may not be applicable to other settings, adaptation takes into account the cultural and organizational differences in the new setting to guarantee their applicability [1]. This requires a well–structured adaptation methodology that takes into consideration affordability and availability of resources and services that would allow contextualizing global guidelines to countries of varying levels of income [2].

A number of adaptation methodologies have been proposed, including the Systematic Guideline Review Method [3], Making GRADE the Irresistible Choice (MAGIC) [4], GRADE–ADOLOPMENT [5], and ADAPTE [1]. GRADE is short for Grading of Recommendations Assessment, Development and Evaluation [6]. ADAPTE is one of the earliest systematic frameworks to adapt guidelines to a local context [1]. The ADAPTE framework consists of 24 steps in three main phases: (1) set–up (preparation), (2) adaptation and (3) finalization [7]. The evaluation of guideline adaptation has been recommended to ensure the reproducibility and efficiency of the methods in producing high quality guidelines [8].

In a recently conducted study, we systematically evaluated the reporting of processes employed in the national adaptation of World Health Organization (WHO) guidelines for Human Immunodeficiency Virus (HIV) and Tuberculosis [9]. We found that 32 out of 170 (19%) guideline adaptations reported their processes. It remains unclear to what extent these findings apply to fields other than HIV and tuberculosis, or to guideline adaptions published in the peer reviewed literature. This is particularly relevant as the adaptation methods used could affect the quality, trustworthiness and applicability of the adapted recommendations.

Our main objective was to describe and assess the methods used for adapting health–related guidelines published in peer–reviewed journals, and to assess the quality of the resulting adapted guidelines.

## METHODS

### Definitions

We adopted the WHO definition of guidelines as “systematically developed evidence–based statements which assist providers, recipients and other stakeholders to make informed decisions about appropriate health interventions” [10]. We considered the following definition of guideline adaptation that is based on the ones proposed by Fervers et al. [1] and by the GRADE–ADOLOPMENT methodology [5]: systematic approach for considering the endorsement or modification of recommendations produced in one setting for application in another as an alternative to de novo development.

### Eligibility

We included documents meeting all the following eligibility criteria:

- Meet the above definition of guideline adaptation;
- Adaptations of specific health–related guidelines (e.g., management of asthma, screening mammography); and
- Published in any language;

We also acquired papers that the authors referred to when describing their adaptation methods, and considered them in the review process.

We excluded documents:

- Describing an adaptation methodology process but not an actual adaptation;
- Meeting abstracts;
- Restricted to implementation only (e.g., for clinical decision support);
- Reporting appraisals of clinical practice guidelines (CPGs) for the purpose of adaptation; or
- Reporting only algorithms.

### Search strategy

We used the OVID interface to search Medline and Embase databases. The period of the search was from January 2000 to June 2015. **Online Supplementary Document(Online Supplementary Document)** provides the detailed search strategies for each database. Also, we searched the reference lists of relevant papers (eg, reviews on adaptation methods) identified in our search. We did not use any language restrictions.

### Selection process

All reviewers underwent calibration exercises. They screened the same set of papers and received feedback on their performance. Then, teams of two reviewers independently screened abstracts and full texts of identified citations for potentially eligible guideline adaptations. Next, the review teams screened the full text of citations judged as potentially eligible by at least one of two reviewers using standardized screening forms. The two members compared their results and resolved disagreement by discussion or with the help of a third reviewer as needed. When excluding an adapted guideline, we recorded the reason for exclusion.

### Data abstraction process

All reviewers underwent calibration exercises for data abstraction. Teams of two reviewers worked in duplicate and independently to abstract relevant information from the included adapted guidelines (except 122 non–English papers which were abstracted by only one person: 63 were in either French or Spanish in which one of the reviewers (RAA) was fluent, and 59 were translated using Google Translate). They used standardized online data abstraction forms on REDCap^TM^ [11]. They compared results and resolved disagreements by discussion, or with the help of a third reviewer.

We abstracted the following characteristics from each included adapted guideline:

- Characteristics of the adapted guideline: name and year of publication, country, contributors to guideline adaptation (governmental body, WHO Headquarter, Regional or National offices, Not for Profit Organizations (NGOs), professional society, “expert panel”), guideline area, reporting of source guideline.
- Characteristics of the source guideline: number of source guidelines, name and year of publication, country, contributors to source guideline development (governmental body, WHO Headquarter, Regional or National offices, NGOs, and professional society, “expert panel”).
- Other information: disclosure of conflicts of interest, and funding (reporting, source, and role).
- We classified income levels of countries (for both adapted and source guidelines) as per the World Bank classification into high, upper–middle, lower–middle and low income countries.

We also assessed whether the authors explicitly reported using an adaptation method. We considered the following options: ADAPTE or one of its variants, other published adaptation method, or an unpublished adaptation method.

Next, we wanted to explore the specific steps followed in the adaptation process. As we did not identify any validated or standardized tool, we decided to rely on the steps described in ADAPTE (**Table 1**) [7]. We chose ADAPTE because it is a well–structured tool, and represents the most widely used method for guideline adaptation. Thus, we abstracted information about the steps covered in the adaptation methodology from papers reporting on at least one element of the adaptation phase of the ADAPTE process [7]. So, the purpose was not to assess compliance with ADAPTE. Rather, we used ADAPTE to identify standard steps in the adaptation approach.

**Table 1:** Phases and steps of the ADAPTE process

Phases	Steps
**Set–up phase**	1. Establish an organizing committee
	2. Select a topic
	3. Check whether adaptation is feasible
	4. Identify skills and resources needed
	5. Complete set–up tasks
	6. Write protocol
**Adaptation phase**	7. Determine the health questions
	8. Search for guidelines and other relevant documentation
	9. Screen retrieved guidelines
	10. Reduce total number of guidelines if there are more than can be dealt with by the panel
	11. Assess guideline quality
	12. Assess guideline currency
	13. Assess guideline content
	14. Assess guideline consistency (search and selection of studies, links between evidence and recommendations)
	15. Assess acceptability/applicability of the recommendations
	16. Review assessments to aid in decision–making
	17. Select between guidelines and recommendations to create an adapted guideline
	18. Prepare a document that respects the needs of the end users and provides a detailed transparent explanation of the process
**Finalization phase**	19. External review by target users
	20. Consult with relevant endorsement bodies
	21. Consult with developers of source guidelines
	22. Acknowledge source documents
	23. Plan for aftercare of the adapted guideline
	24. Produce high quality final guideline

To appraise the quality of adapted guidelines, we used the Appraisal of Guidelines for Research and Evaluation (AGREE) II instrument [12]. This tool was designed to assess the quality of guidelines by evaluating the rigor of guidelines development and transparency in reporting its processes. It is a 23–item tool, in which the items are distributed into six quality domains: scope and purpose, stakeholder involvement, rigor of development, clarity of presentation, applicability, and editorial independence (**Online Supplementary Document(Online Supplementary Document)** provides a listing of the tool’s 23 items and a description of its domains). The items in each domain are rated on a 7–point scale, where 1 indicates ‘Strongly Disagree’ and 7 indicates ‘Strongly Agree’. We calculated the AGREE II “scaled domain score” for each domain, as suggested by the AGREE II group. For this, we added the scores of individual items of a particular domain, then we scaled the total score as a percentage of the maximum possible score in that domain [13]. The resulting scores of the six domains are independent and are not compared to a minimum score as recommended by the AGREE Consortium [12].

### Data analysis

We conducted a descriptive analysis of all variables. We used frequencies and percentages for categorical variables. For continuous variables, we assessed the distribution for normality using the Kolmogorov-Smirnov test. For non–normally distributed variables, we used median and Inter–Quartile Range (IQR); otherwise we planned to use mean and standard deviation.

We used the Mann-Whitney test to assess the association between reporting (vs not reporting), the use of a published adaptation method and the quality of the adapted guideline, measured as the mean score for each AGREE II domain.

## RESULTS

### Study selection

**Figure 1:**
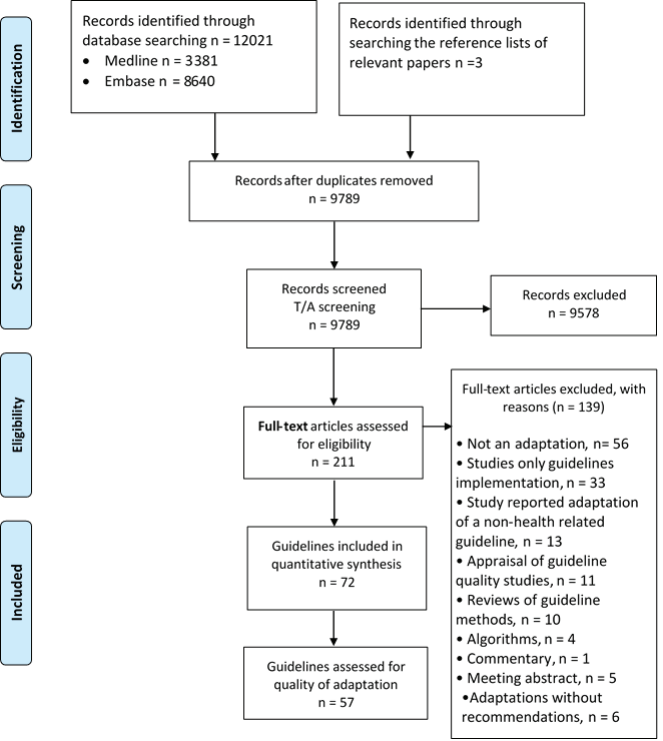
Study flow diagram.

**Figure 1** shows the study flow. Out of a total of 12 021 captured citations, we identified a total of 72 eligible papers, each reporting on one guideline adaptation project. One of those (the Guideline of the German Society for Nutritional Medicine (DGEM)) included 13 different chapters in clinical nutrition but we considered them as one guideline. We excluded 139 papers based on full text screening for the following reasons: 56 were not related to adaptation, 33 were adaptations for implementation only, 13 reported adaptation of a non–health–related guideline, 11 were appraisals for guideline quality, and 10 were review articles, 4 reported algorithms only, 5 were meeting abstracts, 6 were guideline adaptations without reporting of the resulting recommendations, and 1 was a guideline commentary.

### Findings

#### Characteristics of the adapted guidelines

**Table 2** provides a description of the 72 included adapted guidelines. Forty three percent (n = 31) of these adapted guidelines were published between the years 2012–2014. High–income countries produced 83% of the adaptations (n = 60), whereas lower–middle income countries produced only 6% (n = 4). Professional societies developed 71% (n = 51) of the adaptations, while governments were involved in only 11% (n = 8) of adapted guidelines.

**Table 2:** Characteristics and setting of the adapted guideline (n = 72)

		No.	%
**Publication year**	Published 2012–2014	31	43
**Country income**	Low income	1	1
	Lower–middle income	4	6
	Upper–middle income	7	10
	High income	60	83
**Guideline developer**	Governmental body	8	11
	WHO regional/national	0	0
	Non–governmental organization (NGO)	1	1
	Professional society	51	71
	Other	12	22
**Guideline area**	Medical	62	86
	Surgical	5	7
	Psychiatry	3	4.2
	Other	5	6.9
**Specifying source guideline**		65	90
**Disclosure of competing interests**	Organizing committee/Guideline development group	34	48
	Panel members only	4	6
	Not reported	34	48
**Funding**	Not reported	38	53
	Reported as not funded	3	4
	Funded	31	43
**Funding source**	Internally funded	2	3
	Governmental	15	21
	Private–for–profit	12	17
	Private not for profit	4	6

The guideline development group reported conflicts of interests in 38 (53%) of the adapted guidelines. Forty seven percent of adaptations reported on the funding source. The most common funding sources were governmental in 15 (21%) adaptations, followed by private–for–profit organizations in 12 (17%) adaptations. Of the thirty–one adapted guidelines that reported being funded, only six reported on the role of the funder in the adaptation process. While three explicitly reported having no role, the other three reported being involved in: preparing the manuscript, employing an author, and covering costs of meetings.

#### Characteristics of the source guideline

Ninety percent of adapted guidelines reported on the source guideline (**Table 3**). The median number of source guidelines was 2.5 with an IQR of 4 (75^th^ – 25^th^ percentiles being 5 and 1). The IQR for the publication year of the source guidelines was 6 years (75^th^ – 25^th^ percentiles being 2010 and 2004). Eighty five percent of source guideline originated from high income countries. Most common source guideline developers were professional societies (68%), and WHO (13%).

**Table 3:** Characteristics of the source guideline (n = 72)

		No.	%
**Country income**	Low income	0	0
	Lower–middle income	2	3
	Upper–middle income	5	7
	High income	61	85
**Developer**	Governmental body	15	21
	WHO regional/national	9	13
	Non–governmental organization (NGO)	7	10
	Professional society	49	68
	Other	4	6

#### Adaptation method (n = 57)

Out of 72 included papers, 57 reported at least one detail about the adaptation method. Of the 57 adapted guidelines that reported at least one detail of the adaptation method, sixty percent (n = 34) did not report using a published method for guideline adaptation. Forty percent (n = 23) reported using an adaptation method: either ADAPTE (n = 13); ADAPTE as modified by the authors (n = 3); or some other previously published method (n = 7), including: the Practice Guideline Evaluation and Adaptation Cycle (n = 4), the Systematic Guideline Review Method (SGR) (n = 1), RAND consensus method (n = 1), and Registered Nurses' Association of Ontario (RNAO) Toolkit for Implementation of Clinical Practice Guidelines (n = 1). As planned, we collected for these 57 papers information about the adaption method (ADAPTE steps) and the quality of the adapted guideline (AGREE II score).

#### ADAPTE steps

The total number of steps of ADAPTE reported to be followed by each of the 57 adapted guidelines ranged between 6 and 22 (out of a maximum value of 23). The median number was 14 and IQR was 4 (75^th^ – 25^th^ percentiles being 17 and 13).

**Figure 2:**
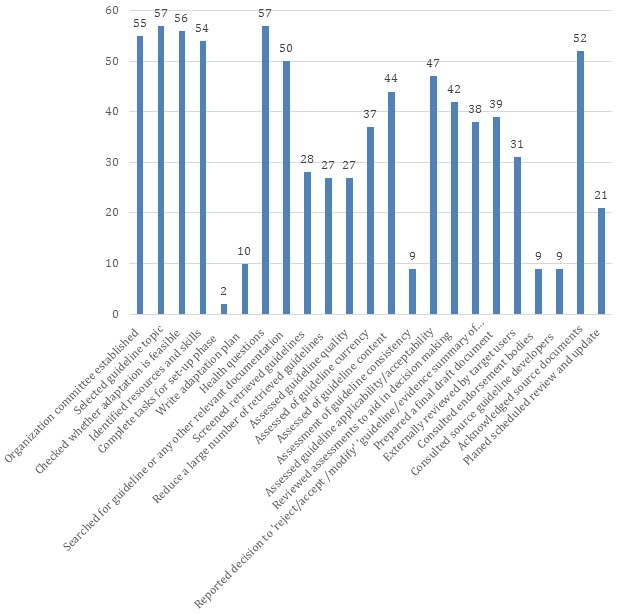
Number of ADAPTE steps fulfilled by adapted guidelines (n = 57).

**Figure 2** show the number of adapted guidelines fulfilling each of the ADAPTE steps. The distribution (out of a maximum value of 57) ranged between 2 (4%) and 57 (100%). The median number was 38 and IQR was 27 (75^th^ – 25^th^ percentiles being 51 and 24). The percentages of guidelines fulfilling ADAPTE steps are presented in **Online Supplementary Document(Online Supplementary Document)**.

At least 95% of the adapted guidelines met the first four steps of ADAPTE (ie, setup or preparatory phase). In the adaptation phase (steps 7 to 18), all adapted guidelines determined health questions, while 88% reported searching for guidelines or any other relevant documents. Only 16% of adapted guidelines assessed guideline consistency ie, assessment of the search and study selection, and links between evidence and recommendation. In the finalization phase: 91% acknowledged the source, but only 16% reported consulting with sources guideline developers.

#### Quality of the adapted guideline (AGREE II score):

**Table 4** reports the AGREE II mean scaled domain scores for the 57 adapted guidelines. The mean scores were highest for the “scope and purpose” domain (93%) and “clarity of presentation” domain (86%). The mean scores were lowest for the two “applicability” and “editorial independence” domains (50% and 43% respectively). The mean score for “rigor of development” was 57%.

**Table 4:** AGREE II mean scaled domain scores for adapted guidelines (n = 57)

Domain	Mean %	Standard deviation	Minimum%	Maximum%	95% confidence Interval
**Domain 1**: Scope and Purpose	93.37	10.09	55.56	100.00	90.69 to 96.05
**Domain 2**: Stakeholder Involvement	64.03	17.22	22.22	100.00	59.46 to 68.60
**Domain 3**: Rigor of Development	56.79	24.85	12.50	100.00	50.20 to 63.39
**Domain 4**: Clarity of Presentation	85.57	25.08	0	100.00	78.91 to 92.3
**Domain 5**: Applicability	50.14	28.85	0	100.00	42.48 to 57.80
**Domain 6**: Editorial Independence	42.54	34.88	0	100.00	33.28 to 51.80

With regards to evaluating the association between reporting the use of a published adaptation method and the quality of the adapted guideline, we found a statistically significant association for the applicability domain but not for the scope and purpose, stakeholder involvement, rigor of development, clarity of presentation and editorial independence domains. For the applicability domain, the scores were 64% when reporting the use of a published adaptation method and 41% when not reporting such use (*P* = 0.005).

## DISCUSSION

### Summary of findings

Our aim was to assess the methods used for adaptation and to assess the quality of health–related guideline adaptations. We identified 72 adapted guidelines published in the past 15 years through an electronic database search. Of the identified guidelines, the majority of both adapted guidelines and their source guidelines were published by professional societies, and in high–income countries. About a fifth of the adapted guidelines did not report any detail about their adaptation methodology. Of those that did, most did not use a published adaptation method. The ADAPTE framework was the most frequently used method but was used only in a quarter of the adapted guidelines. It is important to note that ADAPTE was developed and published after the publication of many of guidelines included in this study. The use of the different steps of guideline adaptation, as well as the quality of the guidelines was variable. A key step before adapting a guideline/recommendation should be an evaluation of how well the source guideline assessed, interpreted and made recommendations, but this may not be occurring. The use of a published adaptation method was associated with a higher score on applicability. This association suggests that the use of adaptation methodologies strengthens the relevance of the adapted guideline.

It is interesting that guideline adaptations are mostly published in high–income countries. It is very likely that adaptations of guidelines in low–income countries are not being reported in peer–reviewed journals, or are being reported in peer–reviewed journals that are not indexed. Capturing those guidelines would require searching governments’ websites as well as national or regional journals.

### Strengths and limitations

The main strength of this study is the use of standard systematic review methodology such as duplicate methods for guideline selection and data abstraction. Also, we used AGREE II instrument, a validated tool for assessment of guideline quality. While AGREE II was not specifically designed for adapted guidelines, we believe it still applies. While we used the ADAPTE steps to assess the process of guideline adaptation, the tool was actually developed for guiding the adaptation process and not for assessing it. Also, this tool was developed and published after the publication of many of adapted guidelines included in this study. Unfortunately, no tool for assessing the adaptation process is currently available. The main limitations relate to restricting the search to electronic databases, and not including adapted guidelines published only in governmental databases or websites, or published locally as reports.

### Comparison to similar studies

We are not aware of any study that systematically evaluated the quality of adapted guidelines. Miguel–Garcia et al. assessed qualitatively the quality of the Spanish adaptation of the European Guidelines on Cardiovascular Disease Prevention in Clinical Practice [14]. The authors highlighted the importance of considering clinical evidence both in developing the source guidelines and in adapting them. Alonso–Coello et al. systematically reviewed studies that used AGREE instrument to appraise guidelines in general (ie, not necessarily adapted guidelines). They found that, despite the fact that quality of guidelines improved over the last two decades, it remained moderate to low when measured with the AGREE instrument [15].

We are aware of two methodological surveys that systematically evaluated guideline adaptation process. Fervers et al. published a literature review in 2006 that identified 18 reports of models, practical examples and experiences of guideline adaptation [1]. They reported that none of these used a validated process for guidelines adaptation. Indeed, that paper was the basis for the ADAPTE framework [7]. Our team conducted the second survey that focused on adaptation of WHO guidelines for HIV and Tuberculosis, and also assessed the number of ADAPTE steps met by the adaptation processes on the national level [8]. The median number of ADAPTE steps in the study of WHO adapted guidelines was 11.5 [IQR = 3.5 (75^th^ – 25^th^ percentiles being 13.5 and 10)] compared to 14 [IQR = 4 (75^th^ – 25^th^ percentiles being 17 and 13)] in the current study. This lower number amongst WHO adapted guidelines was mainly related to lower values for the ‘adaptation phase’. The difference could be related to the fact that guidelines assessed in the current study underwent peer review, which might have improved their reporting, or led to the selection of those with better processes.

### Implications for practice

Guideline adaptation projects need to improve the reporting of their methods. This work suggests that when adapting guidelines, developers have either not evaluated the consistency of the source guideline (links between evidence and recommendation), or have not reported that they did. Increasing awareness of the different phases of adaptation, providing additional tools to facilitate evaluation, facilitating collaboration between developers, or making the evidence to recommendation process more transparent, may be warranted. Guideline adaptation developers also need to follow methodologies specifically designed for this purpose. For example, a large number of guidelines are currently using the GRADE methodology. Also certain adaption methodologies, eg, the “GRADE–ADOLOPMENT” framework encompasses adoption, adaptation and de novo guideline development. This process is building on newly–developed but also already published systematic reviews and health technology assessment reports. Another methodological advancement is the use of the GRADE EtD frameworks, which could facilitate the adaptation process [16].

### Implications for future research

There is a need to develop a standardized tool for assessing the quality of conduct and of reporting of guideline adaptations, given that this methodology has features that are distinct from guideline development. Such a tool would be helpful for both researchers in the field of guideline adaptation, and groups working on adapting guidelines. Also, research focusing on non–peer reviewed guidelines is needed to better assess methods used for adaptation efforts in low and middle–income countries (LMIC).
