# Supplementary material for: Methods used in adaptation of health–related guidelines: A systematic survey
Source: J Glob Health. 2017 Dec 22;7(2):020412. doi: 10.7189/jogh.07.020412 (PMC5740392; doi:10.7189/jogh.07.020412)
Supplement: Online Supplementary Document [file jogh-07-020412-s001.pdf]

Online Supplementary Document

Abdul–Khalek et al. Methods used in adaptation of health related guidelines: A systematic survey

J Glob Health 2017;7:020412

Appendix S1: Search strategy

Database: Embase <1996 to 2015 February 13>, Ovid MEDLINE(R) In-Process & Other Non-Indexed Citations, Ovid MEDLINE(R) Daily and Ovid MEDLINE(R) <1946 to Present>

Search Strategy:

----

1 exp Guideline/ (25868)

2 guidelines as topic/ or practice guidelines as topic/ (355350)

3 (guideline* or guidance or consensus).ti. (156468)

4 exp practice guideline/ (336210)

5 exp consensus development/ (13711)

6 (adapted or adapt or adapts or adaptation or adaption or adapte).tw. (386469)

7 1 or 2 or 3 or 4 or 5 (523585)

8 6 and 7 (6684)

9 limit 8 to yr="2000 -Current" (6139)

****

Figure S1: Percentages of ADAPTE steps fulfilled by adapted guidelines (n=57)

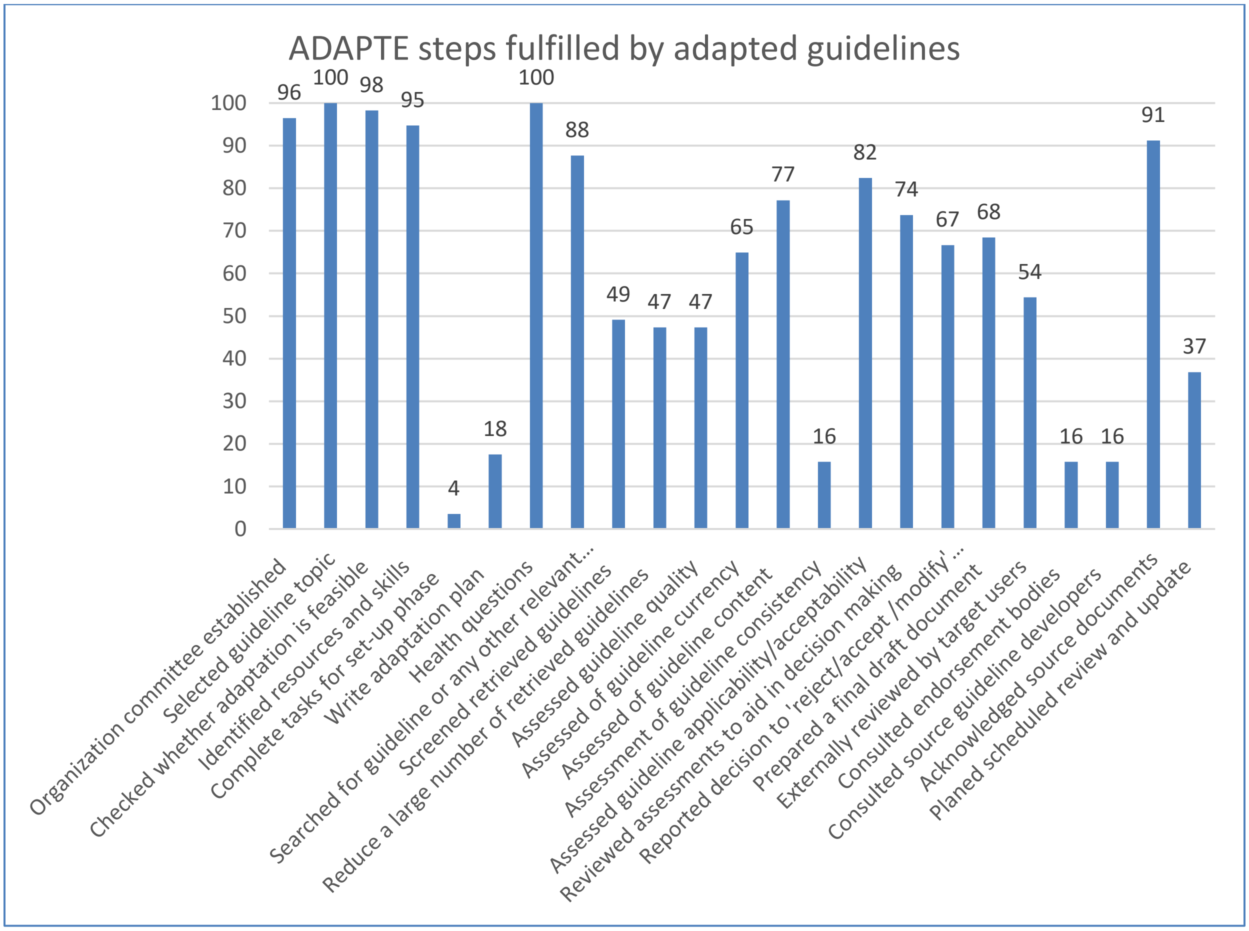

Appendix S2: AGREE-II domains with the respective items in each domain, used for the assessment of the quality of the adapted guidelines.

Description of AGREEE II domains:

1. The domain ‘scope and purpose’, look at the overall aim of the guideline, health questions and target population.
2. ‘Stakeholder involvement’ is concerned with extent to which the guideline was developed by the appropriate stakeholders and represents the views of its intended users
3. ‘Rigor of development’ relates to the process used to gather and synthesize the evidence, the methods to formulate the recommendations, and to update them
4. ‘Clarity and presentation’ deals with the language, structure, and format of the guideline.
5. ‘Applicability’ pertains to the likely barriers and facilitators to implementation, strategies to improve uptake, and resource implications of applying the guideline
6. ‘Editorial independence’ is concerned with the formulation of recommendations not being unduly biased with competing interests

Listing of AGREE II items

Domain 1: Scope and purpose

1. The overall objective(s) of the guideline is (are) specifically described.
2. The health question(s) covered by the guideline is (are) specifically described.
3. The population (patients, public, etc.) to whom the guideline is meant to apply is specifically described.

Domain 2: Stakeholder involvement

4. The guideline development group includes individuals from all relevant professional groups.
5. The views and preferences of the target population (patients, public, etc.) have been sought.
6. The target users of the guideline are clearly defined.

Domain 3: Rigor of development

7. Systematic methods were used to search for evidence.
8. The criteria for selecting the evidence are clearly described.
9. The strengths and limitations of the body of evidence are clearly described.
10. The methods for formulating the recommendations are clearly described.
11. The health benefits, side effects, and risks have been considered in formulating the recommendations.
12. There is an explicit link between the recommendations and the supporting evidence.
13. The guideline has been externally reviewed by experts prior to its publication.
14. A procedure for updating the guideline is provided.

Domain 4: Clarity and presentation

15. The recommendations are specific and unambiguous.

16. The different options for management of the condition or health issue are clearly presented.

17. Key recommendations are easily identifiable.

Domain 5: Applicability

18. The guideline describes facilitators and barriers to its application.

19. The guideline provides advice and/or tools on how the recommendations can be put into practice.

20. The potential resource implications of applying the recommendations have been considered.

21. The guideline presents monitoring and/or auditing criteria.

Domain 6: Editorial independence

22. The views of the funding body have not influenced the content of the guideline.

23. Competing interests of guideline development group members have been recorded and addressed.
